# Effect of repetitive transcranial magnetic stimulation on mood in healthy subjects

**DOI:** 10.3402/snp.v6.29672

**Published:** 2016-03-17

**Authors:** Virginie Moulier, Christian Gaudeau-Bosma, Clémence Isaac, Anne-Camille Allard, Noomane Bouaziz, Djedia Sidhoumi, Sonia Braha-Zeitoun, René Benadhira, Fanny Thomas, Dominique Januel

**Affiliations:** Unité de Recherche Clinique, EPS Ville Evrard, Neuilly-sur-Marne, France

**Keywords:** repetitive transcranial magnetic stimulation, dorsolateral prefrontal cortex, emotion, mood, healthy subjects

## Abstract

**Background:**

High frequency repetitive transcranial magnetic stimulation (rTMS) of the left dorsolateral prefrontal cortex (DLPFC) has shown significant efficiency in the treatment of resistant depression. However in healthy subjects, the effects of rTMS remain unclear.

**Objective:**

Our aim was to determine the impact of 10 sessions of rTMS applied to the DLPFC on mood and emotion recognition in healthy subjects.

**Design:**

In a randomised double-blind study, 20 subjects received 10 daily sessions of active (10 Hz frequency) or sham rTMS. The TMS coil was positioned on the left DLPFC through neuronavigation. Several dimensions of mood and emotion processing were assessed at baseline and after rTMS with clinical scales, visual analogue scales (VASs), and the Ekman 60 faces test.

**Results:**

The 10 rTMS sessions targeting the DLPFC were well tolerated. No significant difference was found between the active group and the control group for clinical scales and the Ekman 60 faces test. Compared to the control group, the active rTMS group presented a significant improvement in their adaptation to daily life, which was assessed through VAS.

**Conclusion:**

This study did not show any deleterious effect on mood and emotion recognition of 10 sessions of rTMS applied on the DLPFC in healthy subjects. This study also suggested a positive effect of rTMS on quality of life.

Repetitive transcranial magnetic stimulation (rTMS) is a neuromodulation technique that uses an electromagnetic coil applied to the scalp producing a magnetic field, which either excites or inhibits regional cortical activity, depending on the parameters of its delivery. Soon after its introduction (Barker, Jalinous, & Freeston, 1985), rTMS was investigated for the treatment of psychiatric diseases. Several meta-analyses demonstrated its clinical efficacy for improving mood symptoms (Gross, Nakamura, Pascual-Leone, & Fregni, 2007; Herrmann & Ebmeier, 2006; Schutter, 2009; Slotema, Blom, Hoek, & Sommer, 2010), particularly in treatment-resistant depression (Lam, Chan, Wilkins-Ho, & Yatham, 2008). The most usual rTMS treatment for depression is the administration of high frequency on the left dorsolateral prefrontal cortex (DLPFC) (Slotema et al., 2010). However mechanisms underlying the efficacy of rTMS on mood remain unclear. Thus, several studies investigated the effects of prefrontal rTMS on mood or emotion processing in healthy subjects. First, two open trials suggested that one unique session of rTMS could induce mood effects in healthy volunteers. High-frequency rTMS over the left prefrontal cortex resulted in a decrease in self-rated happiness and an increase of sadness in comparison with the right prefrontal stimulation (George et al., 1996; Pascual-Leone, Catalá, & Pascual-Leone Pascual, 1996). However, other studies using a sham-controlled condition failed to show an effect on mood of such stimulation parameters in healthy subjects (Baeken et al., 2006, 2008, 2014; Mosimann, Rihs, Engeler, Fisch, & Schlaepfer, 2000). In addition, one session of low frequency rTMS on the right or left prefrontal cortex did not cause any mood change in healthy subjects (Grisaru, Bruno, & Pridmore, 2001; Jenkins, Shajahan, Lappin, & Ebmeier, 2002). Therefore, it has not been proven that a single session of rTMS can induce any mood change in healthy subjects. However, a single session might not be sufficient to highlight such an effect. Indeed, Schaller et al. (2011) demonstrated that nine sessions of high-frequency rTMS applied on the left DLPFC induced a significant reduction of the Beck Depression Inventory (BDI) score in healthy subjects after active rTMS compared with sham rTMS. Until now no study has tried to replicate this work. Relative to emotion processing, only one study examined the effects of a single rTMS session applied on DLPFC in healthy subjects: de Wit et al. (2015) showed that a session of low-frequency rTMS over the left DLPFC the left DLPFC did not impair emotion task performances in volunteers. To our knowledge, no study has focussed on the effects of several rTMS sessions on emotion processing. Hence our objective was to determine the effects of multiple sessions of rTMS applied to the left DLPFC on mood and emotion recognition in healthy subjects.

## Methods

### Participants

Twenty healthy right-handed volunteers aged between 18 and 65 years old were recruited in the study. The local ethics committee approved the study. All subjects signed informed consent. Subjects reporting any current or previous psychiatric or neurological disorders, and pregnant women were not included. In order to ensure that the potential subjects met the criteria, they were presented with the Mini-International Neuropsychiatric Interview (Sheehan et al., 1998), the 13-item BDI (Beck, Rial, & Rickels, 1974), and the Hamilton Depression Rating Scale (HDRS) (Hamilton, 1960), with a threshold score of eight for BDI and HDRS. Subjects were financially compensated for their participation in the study.

### Study design

A two-arm double-blind randomised trial was conducted. Twenty healthy subjects were randomly assigned to an active rTMS arm (*n*=10) or a sham rTMS arm (*n*=10).

### Assessments

At baseline and after 2 weeks of active or sham rTMS, patients were assessed with the BDI, the HDRS, and the Mania Assessment Scale (MAS) (Bech, Rafaelsen, Kramp, & Bolwig, 1978). To further investigate subjects’ self-perception of mood, visual analogue scales (VASs) were used exploring seven dimensions: anxiety, sadness, anger, happiness, nervousness, serenity, and adaptation to daily life. VASs were performed with a 7-cm horizontal line indicating ‘more than usual’ on the left end and ‘less than usual’ on the right end. The Ekman 60 faces test was used to test recognition of facial expressions of basic emotions: anger, disgust, fear, happiness, sadness, and surprise (FEEST, Young, Perrett, Calder, Sprengelmeyer, & Ekman, 2002). There were 10 examples of facial expressions of each emotion, leading to a score out of a maximum of 60 for overall performance, or scores out of 10 for recognition of the six emotions.

### rTMS

A Magstim Super Rapid stimulator (Magstim Company, Whitland, Wales) was used. Stimulation was delivered on the left DLPFC with the following parameters: twenty-five 8-s trains of 10 Hz; 30-s inter-train intervals; 2,000 pulses/session; 110% of the resting motor threshold, defined as the lowest stimulation intensity, at which 5 out of 10 TMS pulses would produce a visible response in the abductor pollicis brevis muscle of the right hand (Rossini et al., 1994). Mean individual motor threshold was 53.67% (3.67). In total, 10 rTMS sessions were programmed every workday for 2 weeks. The TMS coil (70 mm double air film coil) was positioned on the left DLPFC through neuronavigation with Brainsight Software (version 1.7.6; Rogue Research Inc, Canada) based on the anatomical MRI of each subject. The left DLPFC was defined as the middle part of the middle frontal gyrus. On the axial section of each individual's MRI, the middle frontal gyrus was identified as the gyrus bounded medially by the superior frontal sulcus, which was located as the perpendicular sulcus to the precentral sulcus (Nauczyciel et al., 2011). For the control group, a 70-mm double air film sham coil providing the same acoustic sensation and visual impact as the active coil was used. The sham coil stimulated the skin and the muscle overlaying the scalp, giving the subjects the sensation of magnetic stimulation.

### Data analysis

Statistical analyses were performed with SPSS^®^17 Software (Chicago, IL). Demographic features such as age and standard of education were compared between the two groups with a student's test for independent samples. A mixed-design analysis of variance (with time condition –before/after rTMS– as within-subject factor and group –active/sham– as a between-subject factor) was performed to analyse mood variables and facial expression recognition scores only when the equality of variances (Levene's test) and the normal distribution of the data (Kolmogorov–Smirnov's test) were ascertained. When one of these last two tests was significant, non-parametric tests were implemented: delta scores (before rTMS minus after rTMS) for the subjects in the active group were compared with those of the sham group with Mann–Whitney's test.

## Results

### Demographic data

The subjects of the active group (three females and seven males) had an average age of 33.7 years old (SD=12.2) and an average education level of 12.8 years (SD=2.8). The subjects of the control group (five women and five men) had an average age of 30.3 years old (SD=8.0) and an average education level of 13.8 years (SD=2.9). No significant differences were found between the two groups for age (*t=*0.74; *p=*0.47) and education level (*t=*0.78; *p=*0.44).

### Mood measures and facial expression recognition test

**Fig. 1:**
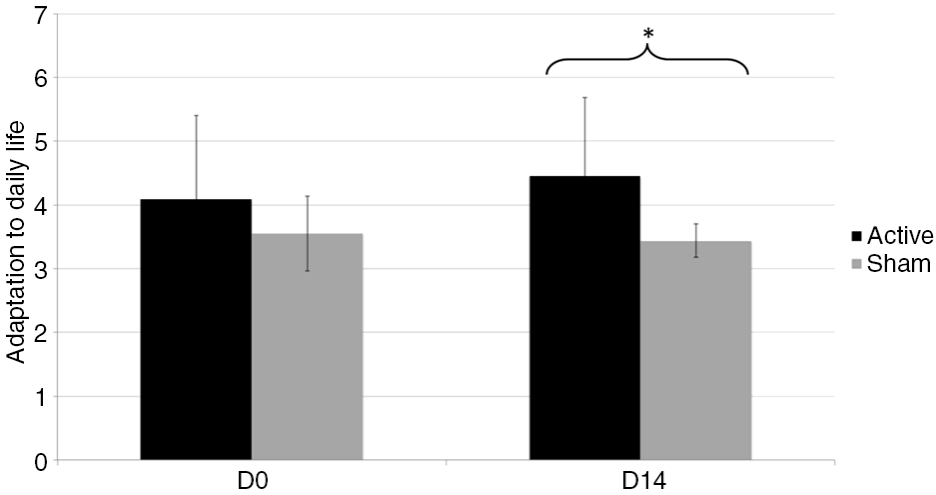
Change in adaptation to daily life quantified by the visual analogue scale before (Day 0) and after (Day 14) 10 rTMS sessions. Notes: The graph shows means and standard deviations; D0=Day 0; D14=Day 14; * *p<*0.05.

Clinical scales (BDI, HDRS, MAS), VASs, and Ekman 60 faces test scores are reported in Table 1. BDI and HDRS data means have been published for a sub-sample of participants (Gaudeau-Bosma et al., 2013). Ten rTMS sessions on the left DLPFC were well tolerated; none of the subjects reported any adverse side effects, except for one subject who suffered from a headache after one session. No subject presented manic or depressive symptoms after rTMS. Statistical analyses of clinical measures, VASs, and Ekman 60 faces test scores are reported in Table 2. No significant group-by-time interaction effect was observed, except for adaptation to daily life (*U=*22.5; *p=*0.036). A trend was found for the perception of anger (*F*_(1,18)_=4.22, *p=*0.056). Concerning the VAS estimating the adaptation to daily life (Fig. 1), there was no difference between groups before rTMS (*U=*49; *p=*0.971). On the other hand, after rTMS, the score was higher in the active group compared with the sham group (*U=*19; *p=*0.019). Adaptation to daily life change was not correlated to mood changes assessed with BDI (ρ=−0.017, *p=*0.942), HDRS (ρ=−0.034, *p=*0.886), and MAS (ρ=0.176, *p=*0.458). Concerning the VAS estimating the perception of anger, there was no difference between groups before rTMS (*U=*39; *p=*0.4) and after rTMS (*U=*37.5; *p=*0.339).

**Table 1:** Clinical assessment, visual analogue scales, and Ekman 60 faces test scores for active and sham groups before and after 10 sessions of rTMS: means, standard deviations (SDs), and 95% confidence intervals

	Active group				Sham group			
		
	Before rTMS		After rTMS		Before rTMS		After rTMS	
				
Variables	Mean (SD)	95% CI	Mean (SD)	95% CI	Mean (SD)	95% CI	Mean (SD)	95% CI
Clinical scales								
BDI	0.40 (0.97)	[−0.29; 1.09]	0.20 (0.42)	[−0.10; 0.50]	0.00 (0.00)	–	0.10 (0.32)	[−0.13; 0.33]
HDRS	0.30 (0.95)	[−0.38; 0.98]	0.70 (1.06)	[−0.06; 1.46]	0.40 (0.97)	[−0.29; 1.09]	0.20 (0.63)	[−0.25; 0.65]
MAS	0.22 (0.44)	[−0.09; 0.53]	0.33 (0.71)	[−0.18; 0.84]	0.25 (0.71)	[−0.26; 0.76]	0.13 (0.35)	[−0.12; 0.38]
Visual analogue scales								
Anxiety	2.59 (1.62)	[1.43; 3.75]	2.19 (1.58)	[1.06; 3.32]	2.58 (1.56)	[1.46; 3.70]	2.93 (0.97)	[2.24; 3.62]
Sadness	2.59 (1.37)	[1.61; 3.57]	2.31 (1.59)	[1.17; 3.45]	2.74 (1.34)	[1.78; 3.70]	2.94 (0.98)	[2.24; 3.64]
Anger	2.80 (1.45)	[1.76; 3.84]	2.24 (1.58)	[1.11; 3.37]	2.40 (1.50)	[1.33; 3.47]	3.00 (1.03)	[2.26; 3.74]
Happiness	4.51 (1.36)	[3.54; 5.48]	4.36 (1.28)	[3.44; 5.28]	3.65 (0.75)	[3.11; 4.19]	3.80 (1.08)	[3.03; 4.57]
Serenity	4.32 (1.40)	[3.32; 5.32]	4.40 (1.30)	[3.47; 5.33]	3.50 (1.03)	[2.76; 4.24]	3.35 (0.17)	[3.23; 3.47]
Nervousness	2.60 (1.49)	[1.53; 3.67]	2.32 (1.63)	[1.15; 3.49]	2.62 (1.64)	[1.45; 3.79]	2.89 (1.02)	[2.16; 3.62]
Adaptation to daily life	4.09 (1.31)	[3.15; 5.03]	4.45 (1.23)	[3.57; 5.33]	3.55 (0.59)	[3.13; 3.97]	3.44 (0.26)	[3.25; 3.63]
The Ekman 60 faces test								
Angry faces	8.40 (1.51)	[7.32; 9.48]	9.30 (0.95)	[8.62; 9.98]	8.10 (1.00)	[7.38; 8.82]	8.10 (1.45)	[7.06; 9.14]
Disgust faces	8.00 (1.16)	[7.17; 8.83]	8.10 (1.66)	[6.91; 9.29]	8.60 (1.65)	[7.42; 9.78]	9.10 (0.74)	[8.57; 9.63]
Fear faces	7.00 (1.89)	[5.65; 8.35]	7.70 (1.64)	[6.53; 8.87]	6.70 (1.49)	[5.63; 7.77]	7.60 (1.71)	[6.38; 8.82]
Happiness faces	9.70 (0.68)	[9.21; 10.19]	9.40 (1.27)	[8.49; 10.31]	9.90 (0.32)	[9.67; 10.13]	9.80 (0.63)	[9.35; 10.25]
Sadness faces	7.50 (1.65)	[6.32; 8.68]	8.00 (2.31)	[6.35; 9.65]	7.50 (1.65)	[6.32; 8.68]	8.30 (1.16)	[7.47; 9.13]
Surprise faces	9.00 (1.25)	[8.11; 9.89]	9.40 (0.70)	[8.90; 9.90]	8.50 (1.35)	[7.53; 9.47]	9.00 (0.82)	[8.41; 9.59]
All faces recognition	49.60 (4.62)	[46.30; 52.90]	51.90 (3.93)	[49.09; 54.71]	49.30 (3.43)	[46.85; 51.75]	51.90 (3.04)	[49.73; 54.07]

Notes: Means (SDs) and 95% confidence intervals are reported for each variable. BDI, Beck Depression Inventory; HDRS, Hamilton Depression Rating Scale; MAS, Mania Assessment Scale; CI, Confidence Interval.

**Table 2:** Results of the statistical analyses for clinical scales, visual analogue scales and Ekman 60 faces test scores

	Effects					
	
	Group		Time		Group×time	
			
Variables	*F*_(1,18)_	*p*	*F*_(1,18)_	*p*	*F*_(1,18)_/*U*	*p*
Clinical scales						
BDI^b^	–	–	–	–	*U*=41.00	0.330
HDRS^a^	0.590	0.452	0.100	0.755	*F*=0.900	0.355
MAS^a^	0.171	0.685	0.002	0.969	*F*=0.456	0.510
Visual analogue scales						
Anxiety^a^	0.366	0.553	0.01	0.921	*F*=2.30	0.150
Sadness^b^	–	–	–	–	*U*=44.50	0.670
Anger^a^	0.091	0.767	0.005	0.944	*F*=4.22	0.056
Happiness^a^	2.761	0.114	0.000	1.000	*F*=0.24	0.630
Serenity^a^	4.261	0.054	0.017	0.898	*F*=0.297	0.593
Nervousness^a^	0.239	0.631	0.001	0.972	*F*=0.902	0.355
Adaptation to daily life^b^	–	–	–	–	*U*=22.50	**0.036**
The Ekman 60 faces test						
Angry faces^b^	–	–	–	–	*U*=30.000	0.111
Disgust faces^b^	–	–	–	–	*U*=42.500	0.544
Fear faces^a^	0.082	0.778	7.945	**0.011**	*F*=0.124	0.729
Happiness faces^b^	–	–	–	–	*U*=49.000	0.914
Sadness faces^b^	–	–	–	–	*U*=47.000	0.815
Surprise faces^a^	1.554	0.228	2.089	0.166	*F*=0.026	0.874
All faces recognition^a^	0.011	0.918	7.050	**0.016**	*F*=0.026	0.873

a When variables met normality and equality of variances assumptions, a mixed-design ANOVA was performed: *F* and *p* values are reported for main effects (group and time) and interaction effect.

b When variables did not meet these assumptions, Mann–Whitney's test compared delta scores (before rTMS minus after rTMS) between the two groups: *U* and *p* values are reported in the last column. Bolded values indicate *p*<0.05.

## Discussion

This double-blind, sham-controlled study did not show any impairment on mood or on facial expression recognition abilities after 10 sessions of 10-Hz rTMS applied on the left DLPFC in healthy subjects, confirming the safety of the technique. This study also showed a positive effect of rTMS on adaptation to daily life, suggesting an improvement of subjective quality of life. This effect of rTMS on quality of life is in accordance with previous findings on patients suffering from depression. Indeed, high-frequency rTMS applied on the left DLPFC (Berlim, McGirr, Beaulieu, & Turecki, 2011; Hadley et al., 2011) and low-frequency rTMS over the right DLPFC (Dumas et al., 2012, 2014) induced a positive effect on quality of life in patients suffering from a major depressive disorder. Interestingly in healthy subjects, subjective quality of life was positively correlated with left prefrontal hemodynamic response, measured through near-infrared spectroscopy (Satomura et al., 2014). Therefore, it seems that prefrontal cortex activity might be linked to subjective quality of life and that the stimulation of this brain area could have an impact on it. However, this potential effect needs to be confirmed in healthy subjects with a validated scale of quality of life in a future study.

With regard to the effect of rTMS on mood measured with clinical scales, our results are in agreement with several studies which failed to show mood effects in healthy subjects after one session of rTMS applied to the prefrontal cortex (Baeken et al., 2006, 2008, 2014; Grisaru et al., 2001; Jenkins et al., 2002; Mosimann et al., 2000). However, our findings differ from the only study which also assessed the effect of several rTMS sessions on mood in healthy subjects (Schaller et al., 2011). Indeed Schaller et al. (2011) found: 1) a significant reduction of BDI score, in a group of 44 healthy subjects after nine sessions of high-frequency rTMS applied on the left DLPFC; and 2) that the active group was more ‘gloomy’ (assessed using a VAS) immediately after the fifth rTMS session. Relative to the BDI, several hypotheses could explain the difference: 1) a possible lack of statistical power, because our sample is smaller than the sample of Schaller et al. (2011); 2) we used a short form of the BDI with 13 items, whereas Schaller et al. (2011) used the 21-item BDI; and 3) our healthy subjects presented lower BDI scores at baseline (mean=0.4 in active group and mean=0.0 in sham group) in comparison with samples of Schaller et al. (2011) (mean=4.4 in active group and 4.0 in sham group).

In our study, the only effect of rTMS on mood was a non-significant trend towards a decrease of anger measured by VAS, probably because VAS is more appropriate for healthy subjects than clinical scales in order to identify subtle variations in mood (Baeken et al., 2008). This potential effect of rTMS applied on the left DLPFC on anger is consistent with recent meta-analyses, which showed that anger was significantly associated with the left DLPFC activity (Kirby & Robinson, 2015; Vytal & Hamann, 2010). However, if the subjective experience of anger tended to change after rTMS, recognition of emotions, including facial expressions of anger, was unaffected in our study. Interestingly, the studies showing an effect of rTMS on the processing of angry stimuli targeted a more medial area: the medial prefrontal cortex (Balconi & Bortolotti, 2013; Harmer, Thilo, Rothwell, & Goodwin, 2001). Thus, it could mean that the subjective experience of anger and the recognition of anger involve different parts of the prefrontal cortex. Nevertheless, our study assessed only emotion recognition based on facial expression. It could be interesting to assess if rTMS applied on the left DLPFC alters skills in emotion recognition from vocal or bodily cues. Additional studies are still required to investigate this potential effect of rTMS on emotion processing.

In future studies, some methodological improvements could optimise the effects of rTMS on mood in healthy subjects. First of all, it would be interesting to adopt a new approach with stimulation of individualised TMS targets in the left DLPFC selected on the basis of their connectivity to deeper limbic regions, as the subgenual cingulate (Fox, Buckner, White, Greicius, & Pascual-Leone, 2012). Furthermore, the accuracy of the motor threshold measurement could be improved using 10 positive motor evoked potentials out of 20 trials instead of 5 out of 10 trials (Rossini et al., 2015). These methodological changes could help improve the reproducibility of the results.

In summary, this randomised sham-controlled study did not show deleterious effects on mood or facial expressions recognition abilities after 10 sessions of 10-Hz rTMS on the left DLPFC and suggested that rTMS could have a positive effect on quality of life in healthy subjects.

## Financial Disclosures

The authors declare no financial interests or potential conflicts of interests with respect to the authorship and/or publication of this article.
